# *GAD1* mRNA Expression and DNA Methylation in Prefrontal Cortex of Subjects with Schizophrenia

**DOI:** 10.1371/journal.pone.0000809

**Published:** 2007-08-29

**Authors:** Hsien-Sung Huang, Schahram Akbarian

**Affiliations:** 1 Graduate School of Biomedical Sciences, University of Massachusetts Medical School, Worcester, Massachusetts, United States of America; 2 Department of Psychiatry, Brudnick Neuropsychiatric Research Institute, University of Massachusetts Medical School, Worcester, Massachusetts, United States of America; University of Munich, Germany

## Abstract

Dysfunction of prefrontal cortex in schizophrenia includes changes in GABAergic mRNAs, including decreased expression of *GAD1*, encoding the 67 kDa glutamate decarboxylase (GAD_67_) GABA synthesis enzyme. The underlying molecular mechanisms remain unclear. Alterations in DNA methylation as an epigenetic regulator of gene expression are thought to play a role but this hypothesis is difficult to test because no techniques are available to extract DNA from *GAD1* expressing neurons efficiently from human postmortem brain. Here, we present an alternative approach that is based on immunoprecipitation of mononucleosomes with anti-methyl-histone antibodies differentiating between sites of potential gene expression as opposed to repressive or silenced chromatin. Methylation patterns of CpG dinucleotides at the *GAD1* proximal promoter and intron 2 were determined for each of the two chromatin fractions separately, using a case-control design for 14 schizophrenia subjects affected by a decrease in prefrontal *GAD1* mRNA levels. In controls, the methylation frequencies at CpG dinucleotides, while overall higher in repressive as compared to open chromatin, did not exceed 5% at the proximal *GAD1* promoter and 30% within intron 2. Subjects with schizophrenia showed a significant, on average 8-fold deficit in repressive chromatin-associated DNA methylation at the promoter. These results suggest that chromatin remodeling mechanisms are involved in dysregulated GABAergic gene expression in schizophrenia.

## Introduction

Cortical dysfunction in schizophrenia and related disease is associated with changes in GABAergic circuitry [1], including altered expression of the 67 kDa isoform of glutamic acid decarboxylase (GAD_67_), one to two key enzymes for GABA synthesis in cortical interneurons. To date, at least 12 studies using tissues from 6 independent brain collections reported downregulated expression for GAD_67_ [2]. In addition, in elderly schizophrenia subjects, up-regulation of GAD_67_ levels was observed [3]. Two lines of evidence point to an important role for GAD_67_ in the neurobiology of schizophrenia: First, dysregulated GAD_67_ expression in the chandelier subtype of GABA neurons is thought to result in disruption of synchronized cortical activity and impairment of working memory functions in schizophrenia subjects [4]. Second, allelic polymorphisms within *GAD1*, the gene encoding GAD_67_, confer genetic risk for childhood-onset schizophrenia and accelerated loss of frontal gray matter [5], [6].

Given the importance of GAD_67_ for the pathophysiology of schizophrenia, it will be important to gain further insight into the molecular mechanisms that underly the reported *GAD1* mRNA alterations in cerebral and cerebellar cortex of schizophrenia subjects [7], [8]. Here we study the potential role of CpG dinucleotide methylation, which at sites of proximal gene promoters often functions as negative regulator of transcription [9]. Recently, studies on prefrontal cortex of schizophenia subjects identified a number of genes, including *REELIN* *[10]* *, COMT* [11] and *SOX10* [12] which are affected by altered DNA methylation in conjunction with changes in mRNA levels. Furthermore, it has been suggested that in psychosis the DNA maintenance methyltransferase enzyme, *DNMT1*, is overexpressed in cerebral cortex [13]. Based on these findings, decreased *GAD1* mRNA expression in interneurons of schizophrenia subjects would be predicted to be associated with increased DNA methylation. However, it is difficult to test this hypothesis directly given that to date no reproducible technology exists to selectively collect-in postmortem brain tissue-genomic DNA from nuclei of *GAD1* expressing neurons. Here, we present an alternative approach that is based on the finding that in neurons, *GAD1* gene expression is associated with the tri-methylation of histone H3-lysine 4 (H3K4me3) a chromatin mark that defines open chromatin at sites of active transcription [14], [15]. Therefore, we separated open and repressive chromatin from human prefrontal cortex with site-specific anti-methyl-histone specific antibodies, followed by *GAD1* DNA methylation studies for each of the two chromatin fractions separately.

## Results

**Figure 1:**
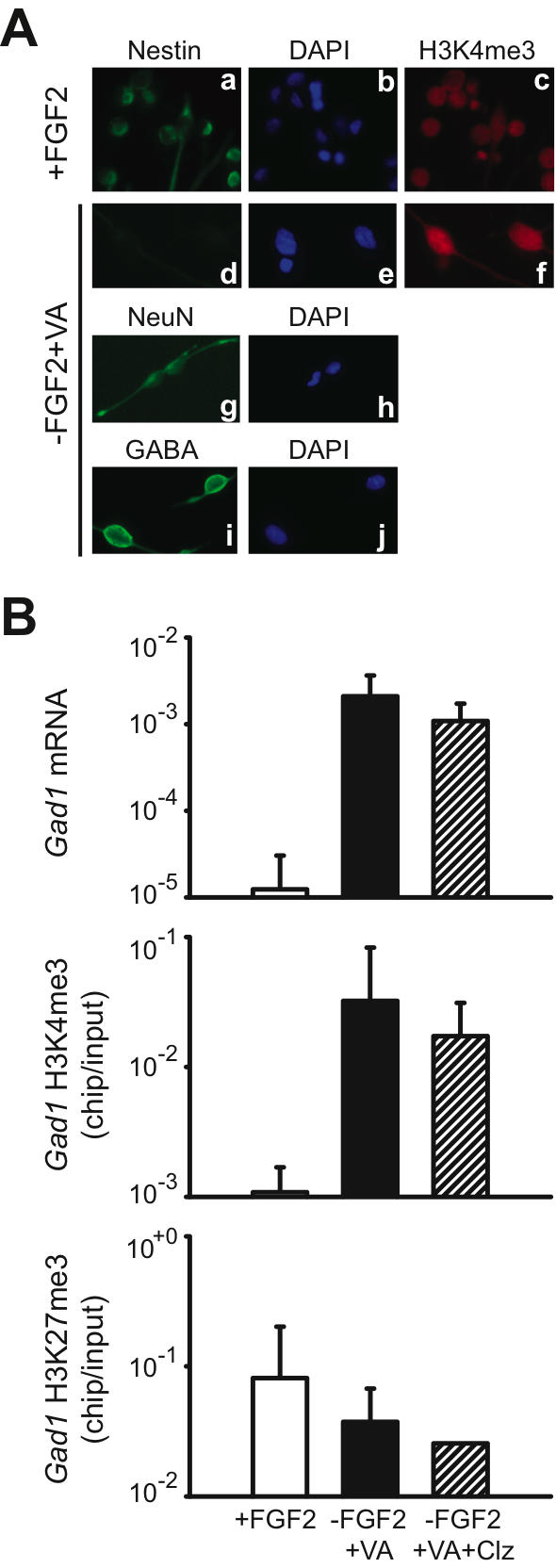
Histone methylation changes at the *Gad1* promoter in a neuronal differentiation assay. (A) (a–j) Digitized images showing (a–c) undifferentiated neural precursors grown in FGF2 (fibroblast growthfactor 2)-containing medium and (d–j) neurons differentiated in medium without FGF2 but with VA (sodium valproate); notice that precursors (a) , but not neurons (d) are defined by nestin immunoreactivity, while both type of cells express robust H3K4me3 immunoreactivity (c,f); (g,i) representative examples of neuronal marker (g, NeuN and i, GABA) immunoreactivity. All images taken at 20×10 magnification. (B) (top) levels of *Gad1* mRNA (y-axis, log scale), expressed relative to *18S* rRNA and (middle and bottom) chip-to-input ratios (y-axis, log scale) of site-specific histone methylation (H3K4me3 and H3K27me3) in the nucleosomes positioned −374 to −273 bp of rat *Gad1* promoter. Data expressed as mean +/− S.E.M., with N = 3 for each of the three different culture conditions. Notice robust increase of *Gad1* mRNA and H3K4me3 levels in differentiated cultures (−FGF2+VA), in comparison to undifferentiated cells (+FGF2); that treatment with the antipsychotic clozapine did not affect histone methylation and *Gad1* gene expression in cultured neurons. VA = Valproic acid, Clz = Clozapine.

To find out if H3K4me3 at the *Gad1* locus defines open chromatin and gene expression, and to examine potential effects of antipsychotic drug (APD) treatment on chromatin remodeling, we monitored open (H3K4me3) and repressive (H3K27me3; (Hampsey and Reinberg 2003; Sims et al. 2003) chromatin-associated histone methylation at the *Gad1* locus in a neural differentiation assay for precursor cells from rat embryonic forebrain (**Fig. 1A**, see also Methods). In this assay, neuronal differentiation is induced by withdrawal of fibroblast growth factor 2 (Fgf2) and addition of sodium valproate (VA) to the cell culture medium [16]. In comparison to undifferentiated precursor cells (“+FGF2” in **Fig. 1A, B**), neurons (“-FGF2/+VA” in **Fig. 1A, B**) showed, on average, a 168-fold fold increase in *Gad1* mRNA levels by qRT-PCR, and this associated with a 30-fold increase in H3K4me3 levels at the proximal *Gad1* promoter (**Fig. 1B**). These changes were consistent in 3/3 experiments. In contrast to these dramatic increases in *Gad1* mRNA and H3K4me3, levels of H3K27me3–the repressive mark–showed a two-fold *decrease* upon neural differentiation (**Fig. 1B**). Furthermore, cultured neurons treated with the antipsychotic, clozapine (“-FGF2+VA+Clz” in **Fig. 1B**), did not show consistent changes in *Gad1* mRNA, or *Gad1*-associated H3K4me3 and H3K27me3. From these experiments, we draw two conclusions: First, the tagging of *Gad1* nucleosomes with H3K4me3-a histone mark previously associated with open chromatin and actual or potential gene expression in non-neuronal tissues and cell lines ([6]–indeed reflects neuronal gene expression activity at that locus. Second, levels of open (H3K4me3) and repressive (H3K27me3) histone methylation at the *Gad1* locus are not affected by treatment with the antipsychotic drug, clozapine.

**Figure 2:**
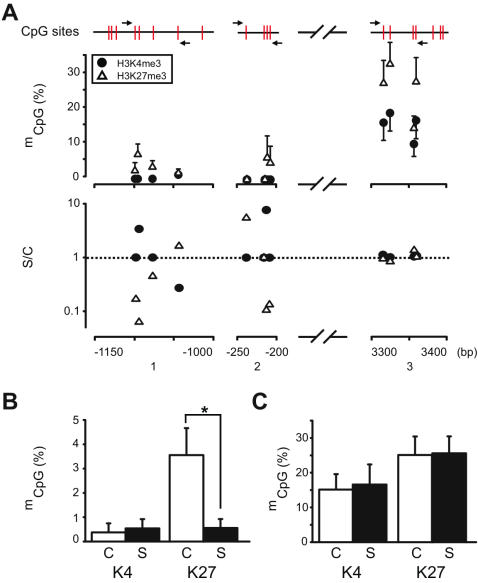
* GAD1* DNA methylation in prefrontal cortex of subjects with schizophrenia and comparison subjects. (*A*) (upper panel) *GAD1* DNA methylation profile in open (H4K4me3) and repressive (H3K27me3) chromatin. Percentage of methylated *GAD1* CpG residues in DNA from immunoprecipitates (y-axis, • , anti-H3K4me3; △ , anti-H3K27me3) for 12 CpG's (shown in red) positioned between bp-1150 to +3400 relative to *GAD1* transcription start site. Primer pairs marked by arrows (*N* = 3–15 control subjects/CpG dinucleotide); (lower panel) averaged levels of methylation for each of the 12 *GAD1* CpG residues (see Fig. 2, upper panel) in schizophrenia subjects (S) , expressed relative to control, C (S/C, y-axis). Notice decreased methylation of 5/8 CpG's at the *GAD1* promoter of schizophrenia subjects (*B,C*) Averaged frequency of DNA methylation at across the 8 CpG's at the *GAD1* (*B*) proximal promoter (see Fig. 2A, upper panel) and (*C*) intron no. 2 in schizophrenia and matched control subjects, as indicated (*N* = 12 clones/primer pair/case or control). Data shown for open (“K4” = H3Kme3) and repressive (“K27” = H3K27me3) chromatin separately. Notice significant deficit in repressive chromatin-associated DNA methylation at the *GAD1* promoter in schizophrenia subjects.

Next, we separated open (H3K4me3) and repressive (H3K27me3) chromatin from postmortem human prefrontal cortex prepared by micrococcal nuclease-based digestion prior to immunoprecipitation as previously described [17], [18] . Then, we monitored *GAD1* mCpG methylation in subclones of PCR products amplified from the immunoprecipitated DNA after bisulfite conversion. Altogether 70 primer pairs within 8kb of *GAD1* proximal promoter and 5′end sequences were tested; 67 primers pairs produced amplicons that lacked sequence specificity (data not shown). This was not surprising given that bisulfite-conversion reduces the genetic code to a 3 letter code in the absence of methylation. The design of suitable PCR primers is further limited by the chromatin preparation technique that produces mononucleosomes with less than 148 bp of genomic DNA. The remaining 3 primer pairs (**Suppl. Table S1**) covered altogether 12 CpG's positioned between −1120 to +3400 bp from the *GAD1* transcription start site (**Fig. 2A**). Methylation frequencies in repressive chromatin immunoprecipiated with anti-H3K27me3 antibody were higher at 10/12 CpG dinucleotides, in comparison to open chromatin fractionated with anti-H3K4me3 antibody (Binomial test, p<0.01) (**Fig. 2A** upper panel). Two CpG's located within 200–250 bp upstream of *GAD1* transcription start site remained unmethylated both in open and repressive chromatin, and DNA methylation levels were overall much lower at the promoter in comparison to intron 2 (**Fig. 2A** upper panel). Next, we monitored *GAD1* CpG methylation levels in subjects diagnosed with schizophrenia and their matched controls. Levels of *GAD1* DNA methylation in open chromatin (H3K4me3) were strikingly similar between schizophrenia subjects and controls, with extremely low levels at the promoter (<0.5%) (**Fig. 2A** lower panel, and **Fig. 2B**) and a higher methylation frequency (approximately 15%) within intron 2 (**Fig. 2A** lower panel, and **Fig. 2C**). Unexpectedly, however, *GAD1* DNA methylation in repressive chromatin (H3K27me3) of schizophrenia subjects was significantly different from control subjects: CpG methylation frequencies were on average 3.5% in the control cohort but only 0.4% in the disease cohort (**Fig. 2A** lower panel, and **Fig. 2B**). This DNA methylation deficit in repressive GAD1 chromatin of schizophrenia subject affected 5/8 GpG nucleotides (**Fig. 2A** lower panel) and was significant (Wilcoxon Signed Ranks Test, p = 0.018). In contrast, the CpG methylation frequencies at intron 2 were very similar in cases and controls (**Fig. 2A**, lower panel, and **Fig. 2C**) and were approximately 25% in both cohorts; these differences were not significant. Therefore, the deficit in prefrontal *GAD1* mRNA levels in this cohort of schizophrenia subjects (**Fig. 2**, see also Methods) is associated with a selective *decrease* in DNA methylation in repressive *GAD1* chromatin at the site of the proximal gene promoter.

Among various *GAD1* single nucleotide polymorphisms (SNP's), two are positioned within 2kb of the transcription start site (*rs3749034* and *rs2270335*). These two SNP's are included in a *GAD1* haplotype that confers genetic risk for childhood-onset schizophrenia and accelerated loss of frontal gray matter [5]. In order to rule out that *GAD1* genotypes were different in the cases and controls of the present study, we determined allele frequencies for the two SNP's. In both cohorts, allele frequencies were identical, which is expected given their close proximity (<1.5 kb) [Schizophrenia subjects, allele (*1/1*) 57%, (*1/2*) 29%, (*2/2*) 14%; controls (*1/1*) 79%, (*1/2*) 14%, (*2/2*) 7%]. Notably, the case and control cohort showed no significant difference in the number of subjects bi-allelic for the common allele (*1/1*), which defines the at risk haplotype [5] (Pearson chi-square *X^2^* = 0.47, df = 1, p = 0.5). Furthermore, overall allele frequencies were not significantly different between the two cohorts (Fisher's Exact Test).

## Discussion

To our knowledge, the present study is the first to assess DNA methylation in human brain separately for open and repressive chromatin. In the open chromatin fractions of the present study, which were defined by trimethylation of a specific histone lysine residue (H3K4) [14], [15], *GAD1* DNA methylation was overall much lower, in comparison to repressive chromatin that is defined by trimethylation of another lysine residue, H3K27 [15]. However, even in repressive/silenced chromatin, only a fraction of *GAD1* CpG dinucleotides (<30% for intron 2, and <5% at the promoter) are subjected to DNA methylation in human prefrontal cortex.

**Figure 3:**
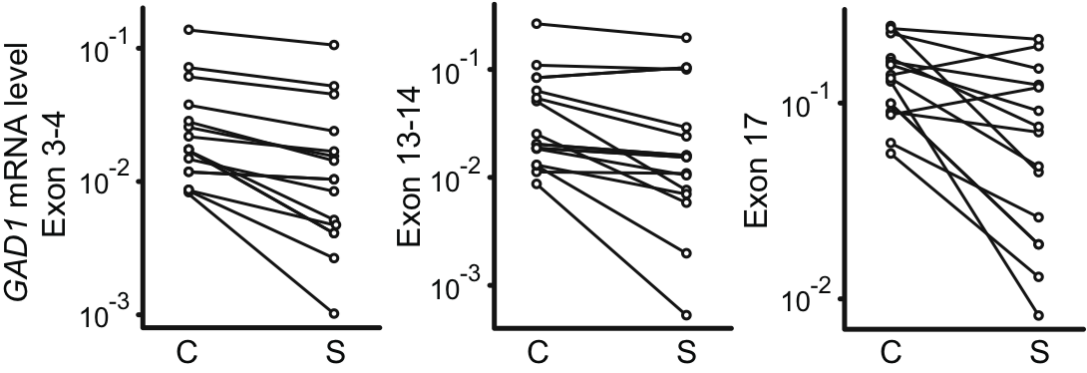
GAD1 mRNA levels in schizophrenia subjects and matched controls. Levels of GAD1 mRNA in 14 controls (C) matched to 14 schizophrenia (S) subjects, as determined by qRT-PCR separately for 3 different primer pairs spanning different exons. Data shown after log-transformation and normalization with housekeeping gene transcript, β2-microglobulin.

**Figure 4:**
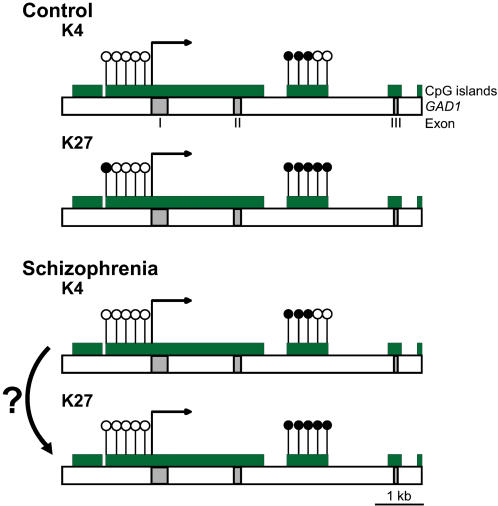
DNA methylation changes at GAD1 locus in schizophrenia. (Top) schematic presentation of *GAD1* CpG dinucleotides (open labels, unmethylated and filled labels, methylated) in normal prefrontal cortex, showing higher levels of DNA methylation in repressive (“K27” = H3K27me3) as opposed to open (“K4” = H3K4me3) chromatin. Notice overall low DNA methylation at proximal *GAD1* promoter. (Bottom) In schizophrenia, *GAD1* promoter DNA methylation in repressive chromatin (“K27”) is further decreased from control values. A hypothetical but plausible mechanisms would be the conversion from open chromatin to repressive chromatin that becomes tagged with the histone mark, H3K27me3, but without additional DNA methylation.

Given that DNA methylation around the proximal promoter typically contributes to transcriptional repression, it was expected that subjects with schizophrenia show increased *GAD1* DNA methylation. Instead, we observed for the repressive chromatin fraction of schizophrenia subjects a significant *decrease* in CpG methylation at the proximal *GAD1* promoter. Further studies are necessary in order to determine if these changes are related to altered *GAD1* gene transcription. Notably, the schizophrenia subjects of the present study had lower *GAD1* mRNA levels in comparison to the matched control (see Methods and **Fig. 3**). One plausible explanation would be that in the affected cases, there is an increased proportion of *GAD1* nucleosomes tagged with the repressive mark, H3K27me3, but without concomitant methylation of the genomic DNA (**Fig. 4**). Therefore, repressive chromatin-associated histone methylation at the *GAD1* locus in schizophrenia appears to be, at least in part, independent from CpG methylation. The present study faces an important limitation because reliable PCR amplification across multiple subjects was achieved only for a dozen *GAD1* CpG's, and we cannot exclude an important role for any of the approximately 400 additional CpG sites that surround the first 5 kb of *GAD1* transcription start site. Therefore, our findings have to be viewed as preliminary. Furthermore, it will be of interest to find out in future studies whether the observed DNA methylation deficits in H3K27me3-tagged *GAD1* nucleosomes of schizophrenia subjects are specific for that gene, or part of a more widespread DNA methylation defect of the disorder.

## Methods

All procedures were approved by the institutional review board of the University of Massachusetts Medical School. Demographics, postmortem confounds and RNA data for the case and control cohorts are presented in **Table 1**. Each pair consisted of a subject with schizophrenia and a control matched for age, gender and autolysis time. The prefrontal cortex of the 14 schizophrenia subjects included in the present study showed, in comparison to the matched control, a decrease in *GAD1* mRNA levels, as determined by qRT-PCR with 3 different sets of PCR primers and normalization to the housekeeping gene transcript, β2-microglobulin (B2M) (**Table 1** and **Fig. 3**). In addition, for 3 of the matched pairs the decrease in *GAD1* mRNA levels in the schizophrenia subject had been observed in a previous study that used in situ hybridization histochemistry [19]. The remaining 11 matched pairs were not included in that earlier study, but were collected by the same brain bank and subjected to the same diagnostic criteria and inclusion/exclusion criteria as previously described [19].

**Table 1:** Postmortem collections

Group	N	Age	PMI	Brain pH	RIN	*GAD1* mRNA			G	APD
						Exon 3–4	Exon 13–14	Exon 17	F/M	
		Mean±S.E.M.								
		years	hrs						No.	%
Cases	14	58.7±5.5	13.6±2.4	6.44±0.09	6.1±0.4	0.023±0.008	0.038±0.015	0.085±0.018	5/9	86
Controls	14	60.5±5.2	13.7±2.5	6.40±0.09	5.6±0.6	0.036±0.010	0.054±0.018	0.143±0.017	5/9	N/A

RIN, RNA integrity number; APD, antipsychotic drug; PMI, postmortem interval

For DNA methylation studies, nucleosomes first were extracted from prefrontal cortex gray matter and then immunoprecipitated with two anti-methyl-histone specific antibodies [anti-histone H3-tri-methyl-lysine 4 (H3K4me3) and anti-H3-tri-methyl-lysine 27 (H3K27me3)] to separate open chromatin at sites of actual or potential transcription from repressive and silenced chromatin, exactly as described [17], [18]. Salmon sperm as a blocking agent was omitted and instead all samples were first pre-cleared by 30 min agarose pre-incubation prior to the immunoprecipitation procedure. DNA purified from immunoprecipitates was subjected to sodium bisulfite conversion followed by purification and elution, using the EZ DNA methylation kit (Zymo research), according to the manufacturer's instructions. Methprimer software (http://www.urogene.org/methprimer) was used to design primer pairs for *GAD1* sequences. Three primer pairs (**Suppl. Table S1**) altogether covering 12 CpG's positioned between −1120 to +3400 bp from the *GAD1* transcription start site, were choosen to profile DNA methylation patterns in control brains (**Fig. 2A** upper panel). PCR amplicons were gel-purified, subcloned into pCR4-TOPO vector, purified in 96 well plates (Qiagen Turbo Miniprep) and for each subject, 12 clones from each immunoprecipitate were analyzed by sequencing. For each subject, DNA methylation levels were expressed as % methylated CpG's separately for each primer pair, and differences between schizophrenia and control subjects were evaluated by two sample t-test , or in case of non-normal distribution, by Mann-Whitney test separately for open and repressive chromatin fractions. Furthermore, for each case and control, genotyping for selected *GAD1* single nucleotide polymorphism was performed using matrix-assisted laser desorption/inonization mass spectrometry (Sequenom), in conjunction with SpecroDesign software for PCR and MassEXTEND primers.

### Cell cultures

Neural stem cells were prepared from forebrain of E14.5 SASCO SD rat embryos (Charles River). Live cells were plated out at 1.2–1.4×10^6^ cells per 100-mm poly-l-lysine coated dishes pre-coated with 15ug/ml poly-l-ornithine (sigma) and 1ug/ml fibonectin (R&D systems), and treated daily with 10ug basic fibroblast growth factor 2 (FGF2-R&D systems). At DIV5, cells were passaged and plated out at 0.8–1.0×10^6^ cells per pre-coated plate and expanded as above for a further 3–4 days (expansion approx 300%). Cells were passaged again and plated out at 1.2–1.4 ×10^6^, and after 1–2 days FGF2 was removed, cells washed once with media and then resuspended in DMEM/M2/F12 media (Invitrogen) without FGF2 but with 0.5 mM sodium valproate and with or without 1 micromol clozapine, and harvested after 4 days.

## Supporting Information

Table S1 (0.03 MB PDF)
