# Supplementary material for: GAD1 mRNA Expression and DNA Methylation in Prefrontal Cortex of Subjects with Schizophrenia
Source: PLoS One. 2007 Aug 29;2(8):e809. doi: 10.1371/journal.pone.0000809 (PMC1950080; doi:10.1371/journal.pone.0000809)
Supplement: Table S1 — (0.03 MB PDF) [file pone.0000809.s001.pdf]

Suppl. Table: Primer for bisulfite-converted *GAD1* DNA (sense strand)

Primer location**	Product* location	Length (bp)	Primer sequence		Amplified strand sequence from bisulfite converted ChIP DNA
			Forward	Reverse	
1	(-1118:-1017)	102	TTGAATTAGTGAGGTTTTAGA	AATAATTCCTTTACTAATATTTACC	TTGAATTAGTGAGGTTTTAGACGTGCGTATAAATAATTTTTGCGTGTTGTATTATTTGGGGAGAGGGGGAGGATTACGGTAAATATTAGTAAAGGAATTATT
2	(-266:-182)	85	GTAGGGTTATAGATTTGGGGGAATTT	CTCCCCCTCCCATATATTATAAAAAAC	GTAGGGTTATAGATTTGGGGGAATTTCGGGGAAAAGTATTGAGGTAAAATCGTCGTTCGTTTTTTATAATATATGGGAGGGGGAG
3	(3289:3378)	90	AATTATTTATATAATAAAGAGGGTTT	CTACCCTAACCAACTCCAAC	AATTATTTATATAATAAAGAGGGTTTCGGGAGAGTCGTTAGTTTTGGTAGTATATGAATTAGGTATCGGCGTTGGAGTTGGTTAGGGTAG

* NT_005403.15, GI: 51461028, Con 19-AUG 2004, 0=21882616=TSS

** Primer location refers to Figure 1

Species Genomic sequences for chromatin immunoprecipitation studies

Species	Gene	Chromosomal location	Product location	Length [bp]	Primer sequence		Source	Gene transcriptiption start site
					Forward	Reverse		
Rat	Gad1	Chr. 2	(-374:-273)	102	TGATCTTTTCCCTGCTGTCA	TCCCATGAGTAATCCAGAACG	NW_047655.1; GI: 34854955; CON 15-APR-2005	21955046
	B2m	Chr. 3	(-477:-367)	111	ACAGGAGGGTTTTTGCACAC	AAAGCCAGGACCATTAAGAACA	NW_047657.2; GI: 62645767; CON 15-APR-2005	50329099

mRNA

Species	Gene	Exon	Product location	Length of [bp]	Primer sequence		Source	Transcript length [bp]
					Forward	Reverse		
Human	GAD1	E3-E4	(676:805)	130	ACTGGGGCTCAAGATCTGC	ATCCCGGTCGCTGTTTTC	NM_000817.1; GI: 4503872; PRI 23-AUG-2004	3610
	GAD1	E13-E14	(1809:1954)	146	AAAAGGGTATACTCCAAGGATGC	CCACATCAGCCAGAACTTGA	NM_000817.1; GI: 4503872; PRI 23-AUG-2004	3610
	GAD1	E17	(3195:3345)	151	ACGCTCTCTGTCTGGCTGTA	AAGGTCTTCGGAAATGTTGC	NM_000817.1; GI: 4503872; PRI 23-AUG-2004	3610
	B2M	E1-E2	(123:272)	150	CCAGCGTACTCCAAAGATTCA	TGCTCCACTTTTTCAATTCTCTC	NM_004048.2; GI: 37704380; PRI 27-OCT-2004	987

mRNA

Species	Gene	Exon	Product location	Length [bp]	Primer sequence		Source	Transcript length [bp]
					Forward	Reverse		
Rat	Gad1	E3-E4	(325:436)	112	GGTTTCTTGCAAAGGACCAA	CACCAGGGTCACTGTTTTCA	NM_017007.1; GI: 8393405; ROD 15-APR-2005	3200
	B2m	E2	(152:253)	102	CAGTTCCACCCACCTCAGAT	GAAAGACCAGTCCTTGCTGAA	NM_012512.1; GI:7549745; ROD: 15-APR-2005	604
	18S rRNA		(1335:1469)	135	CATGGCCGTTCTTAGTTGGT	GAACGCCACTTGTCCCTCTA	X01117.1; GI: 57149; ROD 06-JUN-2003	1874
